# Species composition of shoreline wolf spider communities vary with salinity, but their diets vary with wrack inflow

**DOI:** 10.1002/ece3.9701

**Published:** 2022-12-28

**Authors:** Peter A. Hambäck, Alyssa R. Cirtwill, Magdalena Grudzinska‐Sterno, Alexander Hoffmann, Marie Langbak, David Åhlén

**Affiliations:** ^1^ Department of Ecology, Environment and Plant Sciences Stockholm University Stockholm Sweden

**Keywords:** *Alopecosa*, *Arctosa*, *Fucus*, molecular gut content analysis, *Pardosa*, shoreline fauna

## Abstract

Wolf spiders are typically the most common group of arthropod predators on both lake and marine shorelines because of the high prey availability in these habitats. However, shores are also harsh environments due to flooding and, in proximity to marine waters, to toxic salinity levels. Here, we describe the spider community, prey availabilities, and spider diets between shoreline sites with different salinities, albeit with comparatively small differences (5‰ vs. 7‰). Despite the small environmental differences, spider communities between lower and higher saline sites showed an almost complete species turnover. At the same time, differences in prey availability or spider gut contents did not match changes in spider species composition but rather changed with habitat characteristics within a region, where spiders collected at sites with thick wrack beds had a different diet than sites with little wrack. These data suggest that shifts in spider communities are due to habitat characteristics other than prey availabilities, and the most likely candidate restricting species in high salinity would be saline sensitivity. At the same time, species absence from low‐saline habitats remains unresolved.

## Graphical abstract

A small change in salinity (5‰ vs 7‰) cause total turnover of wolf spider species composition. Wolf spider diets do not vary between species. Wolf spider diets differ between sites with or without a thick wrack bed.

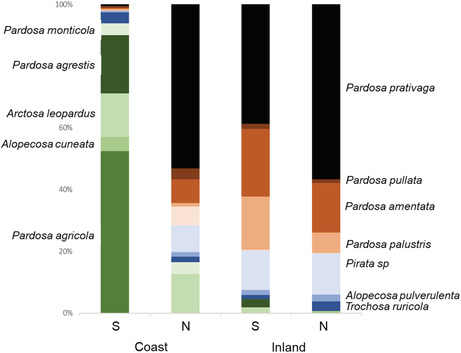

## 1 INTRODUCTION

Shorelines and other riparian habitats are often described as hotspots for arthropod predators, and spiders in particular seem to thrive in these habitats (Batzer & Wu, 2020; Mellbrand & Hambäck, 2010; Polis & Hurd, 1995). There are several reasons underlying these high spider densities, but an important factor seems to be the high prey density in these near‐water habitats (Polis & Hurd, 1995; Sanchez‐Ruiz et al., 2018), which also reduces intraguild predation (Wise, 2006). Prey densities are high in these sites both because of a direct inflow of insects from the aquatic environment, such as midges with aquatic larvae and terrestrial adults, and because large inflows of organic material are deposited on shorelines providing food for detritivores and fertilizing plants (Baxter et al., 2005; Colombini & Chelazzi, 2003; Hyndes et al., 2022). At the same time, most shorelines are harsh environments due to flooding and wave disturbance, and on marine shorelines due to a high salinity and a high turnover of organic material (Barboza & Defeo, 2015; Defeo & McLachlan, 2013). Species diversity on shorelines may therefore be poor, particularly on marine shorelines where communities often consist of a range of habitat specialists that can endure high salinity levels (Cheng, 1976; Irmler et al., 2002).

Despite these general patterns, there is a lack of understanding on how physical processes and prey availability interact in shaping coastal arthropod communities (Hyndes et al., 2022). In fact, the spatial variability of arthropod communities in these habitats is poorly documented compared with inland habitats. For instance, what differences in the species composition between limnic and marine shorelines are due to direct effects from a saline environment and what differences are rather due to differences in prey communities? Prey communities on limnic shorelines are often dominated by midges and a range of other taxa (Benke, 1998; Delettre & Morvan, 2000; Salvarina et al., 2017), whereas prey communities on marine shorelines are more dominated by species developing in rotting wrack beds (Hyndes et al., 2022; Schlacher et al., 2017). Similarly, what is the relative importance of the inflow of dead organic matter versus prey that developed in the water for shoreline predators? Previous studies suggest that the importance of these different resources for spiders and other shoreline predators may vary both between sites, between life stages, and over time (Mellbrand et al., 2011; Paetzold et al., 2008; Verschut et al., 2019). The diet analysis of spiders across the season by Verschut et al. (2019) indicated that adult wolf spiders during early summer on marine shorelines feed largely on terrestrial dipterans such as dung flies, which have developed in wrack beds, whereas juvenile wolf spiders later in season had fed more on aquatic dipterans such as chironomids, where the larvae had fed on algae or detritus in the water.

**FIGURE A1:**
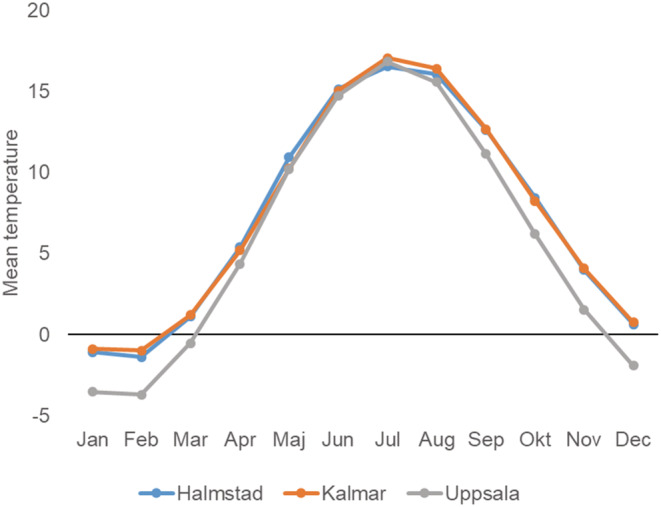
Temperature profiles for the three study regions: south inland (Halmstad), south coastal (Kalmar), and north inland/coastal (Uppsala).

To approach these questions, we studied prey communities, spider diets, and spider community structure between regions with different salinity along the Swedish coast. The salinity changes continuously from freshwater (<1‰) in the inner parts of the Bothnian Bay to oceanic conditions (>30‰) on the western coastlines, which allow us to explore effects from comparatively small salinity differences. In this study, we included two coastal regions with 5 ‰ and 7 ‰, respectively, where previous studies have indicated the shifting dominance of spider species (Hambäck et al., 2016; Verschut et al., 2019). We focus our attention on wolf spiders because these typically dominate the shoreline predator community in the area (Mellbrand & Hambäck, 2010). To account for the role of marine inflow, we aimed to include sites with and without thick wrack beds in each region. We also needed to control for climatic effects because the salinity gradient for our study is also a latitudinal gradient. For this reason, we used a similarly collected data set of spider communities on shores by inland waters along the same latitudinal gradient and with similar climate (Figure A1). Finally, to examine the role of a changing prey community and spider diet, we estimate prey densities using SLAM traps and collected spiders for gut metabarcoding in the same sites. Prey densities and spider diets were estimated two times, to cover seasonal changes in prey availability and diet differences between adult and juvenile spiders (cf. Verschut et al., 2019).

## 2 METHODS

**FIGURE 1:**
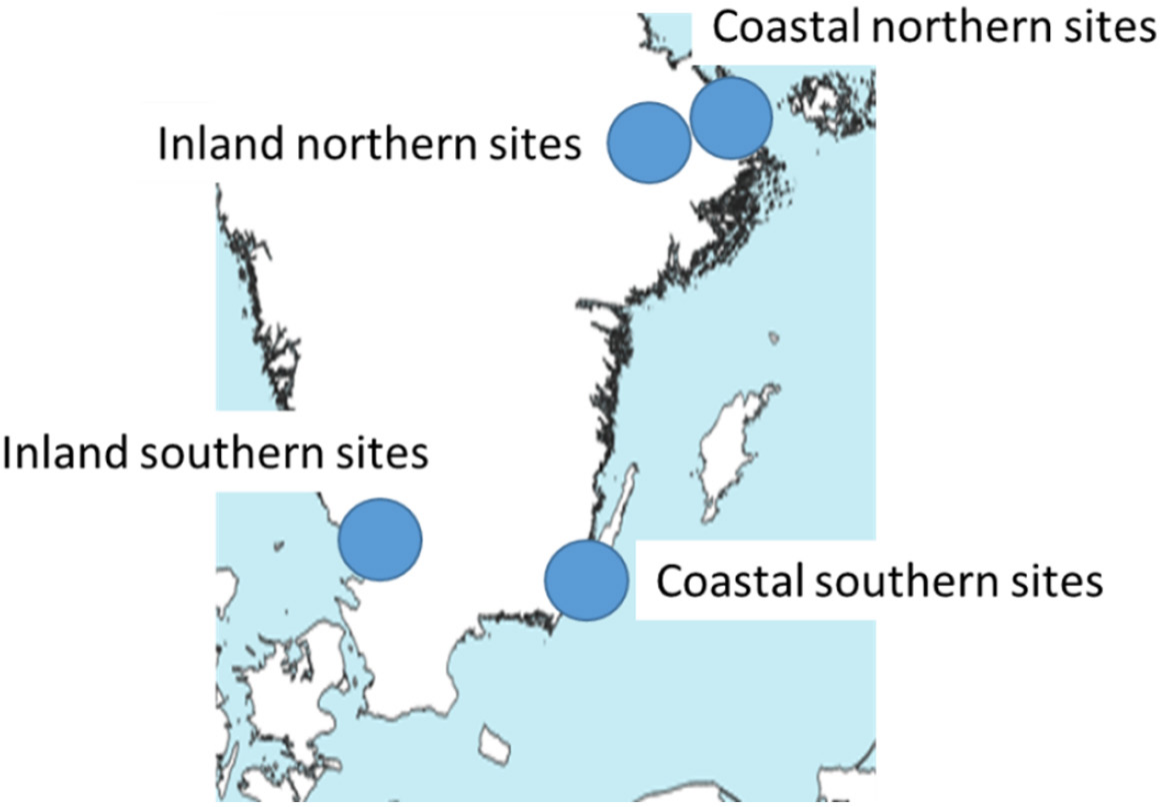
Map showing the location of regions (northern sites = Uppland, southern coastal sites = Kalmar, southern inland sites = Halland). For site information, see SI Table A1.

*Study sites*: The coastal regions included in the study were (a) Uppland north of Stockholm with the lowest salinity (≈5‰, northern region) and (b) Kalmar and Öland in southeastern Sweden with somewhat higher salinity (≈7‰, southern region) (Figure 1, Table A1). The numbers of coastal sites were 13 (Uppland) and 7 (Kalmar). Among these, two sites, respectively, had thick wrack beds, and the other sites were similar but without thick wrack beds and often with short‐cut grass due to grazing. The thick wrack beds had a thickness of more than 20 cm with a considerable extension (several 10 s of meters). The nonwrack sites either lacked wrack almost completely (as in the Uppland region) or that wrack occurred in scattered patches and never so thick as to provide a suitable habitat for detritivores (as in the Kalmar region). The inland regions included 15 and 23 shoreline sites in Uppland and southern Halland (same latitude as Kalmar), respectively (Figure 1; Table A1), as part of a broader study focusing on both insect and spider communities in wetlands.

*Field sampling*: Coastal wolf spider communities were sampled using 10 pitfall traps per site placed in the wrack (wrack sites) or in open ground (nonwrack sites) for three nights in early May 2022 (Kalmar) or early‐mid June 2021 and late May 2022 (Uppland). Inland wolf spider communities were sampled during June 2020 from sites between a few km to more than 100 km inland. These times were chosen because wolf spiders are then adults or subadults, which simplifies species identification, and abundances are quite constant until the end of June when reproduction is finished and adults die off. The interannual differences are also small, as indicated by multiannual trapping campaigns in some of the included sites (Hambäck unpubl. data). Captured spiders were placed in 70% ethanol and brought to the laboratory for identification. Spiders from the inland sites were identified by R. Vicente and those from coastal sites by M. Langbak and A. Hoffmann, with assistance from R. Vicente for complicated cases (mainly involving *Pardosa agrestis/agricola/monticola*).

Spiders used for diet analyses were only collected from 13 sites, six from Uppland and seven from Kalmar, including all wrack sites. Spiders were individually collected by hand (30 per site), to reduce contamination risk, at two times (June and August 2019) and directly transferred to 95% ethanol. In the lab, samples were placed in a freezer (−20°C) until DNA extraction and further processing. Finally, prey densities were estimated by placing one SLAM (Sea Land Air Malaise) trap for two nights at the same time when collecting spiders for diet analyses. SLAM trap catches were placed in 70% ethanol, and brought to the laboratory for sorting to family or order level.

*Diet analyses*: To metabarcode prey content of the hand‐collected spiders, DNA was extracted from either a dissected abdomen (larger spiders) or the whole specimen (small spiders). To reduce the DNA yield of the focal spiders, we used a forward primer designed not to amplify wolf spider DNA (NoSpi2, Lafage et al., 2020) in combination with a general reverse primer (fwhR2n, Vamos et al., 2017) to amplify a section within the Folmer region of COI (Folmer et al., 1994). Procedures for PCR amplification and library building follow Hambäck et al. (2021), and sequencing of the spider samples was performed in one batch on the Illumina MiSeq3 platform at SciLifeLab in Stockholm. To detect individual samples after sequencing, a dual tagging approach was used where the 5′‐end of both primers included an 8 base‐pair tag (Binladen et al., 2007). Illumina‐adaptors bearing unique indices were then ligated to the phosphorylated amplicons without a PCR step to preclude tag‐jumping errors (Bohmann et al., 2022). Due to problems with low DNA content, we had to change the strategy and add a second PCR step with a low cycle number (6). Because this additional step increases the risk of tag‐jumping errors, we built libraries separately for each site, using SMARTer ThruPLEX DNA‐seq library preparation kit excluding fragmentation of DNA (Takara Bio), as tag jumps between spiders within the site do not affect the results due to pooling at this level before analysis. In each library, we also included at least 25% empty combinations to estimate tag‐jumping errors (which was about 6%). After sequencing, we used ObiTools (Boyer et al., 2016) within the Galaxy Platform (Jalili et al., 2020) to assemble paired‐end sequences of high quality (score > 40), trim primers, clean sequences using “obiclean,” and demultiplex resulting sequences to individual samples using “NGSFILTER” after filtering for size. These procedures resulted in a data set of 367 spider individuals and about 384,600 prey sequences that were grouped based on 97% similarity and where representative sequences were taxonomically assigned using BoLD (Ratnasingham & Hebert, 2007) before further analyses.

*Statistical analyses*: Spider communities were modeled as the abundance of each spider species per site in a multivariate analysis with region, inland/coast, wrack, and the region‐by‐inland/coast interactions as independent variables using the command manyglm (package: mvabund, Wang et al., 2012) with a negative binomial error distribution. Prey communities were similarly modeled as the abundance of major groups in a multivariate analysis with manyglm between regions with wrack as an independent variable and a negative binomial error distribution, but these tests additionally included season (June and August) as an independent variable. Finally, the proportional number of prey sequences (logit‐transformed) of major groups were pooled for each species within site and season and was modeled using adonis2 (package: vegan, Oksanen et al., 2019). To compare diet composition between spider species, we also compared gut contents while controlling for the effects of the region. To examine model assumptions, we used plot.manyglm and all tests showed no pattern in errors, which confirms the model appropriateness. Significant relationships were further explored using ANOVA with adjusted *p*‐values, to identify which groups that explained the variation. In all these tests, prey communities and spider diets were included at the level of family or higher taxonomic unit and not at a species level.

To study prey diversity and diet consistency within and among species, we first calculated individual diets using the dynamic threshold model in Cirtwill and Hambäck (2021). We then compared species accumulation curves in spider guts using specaccum with spider individual as a sampling unit (package: vegan, Oksanen et al., 2019) and then estimated diet consistency by calculating the Jaccard similarity index between diets of individual spiders' prey species and prey families, first between pairs of all spider individuals and then between individual pairs of the same species. Diet similarity was compared between region, wrack, and their interaction, firstly, depending on if pairs included all spider individuals or were restricted to within species comparison and, secondly, depending on if diets were based on prey species or prey family. If the interaction terms did not contribute, models were re‐fit without the interaction. We then tested for pairwise differences between region‐wrack combinations using a Tukey's HSD test applied to the analysis of variance of the above linear models, including the interaction term between region and wrack. All tests were performed using R 3.6.3 (R Core Team, 2020).

## 3 RESULTS

**FIGURE 2:**
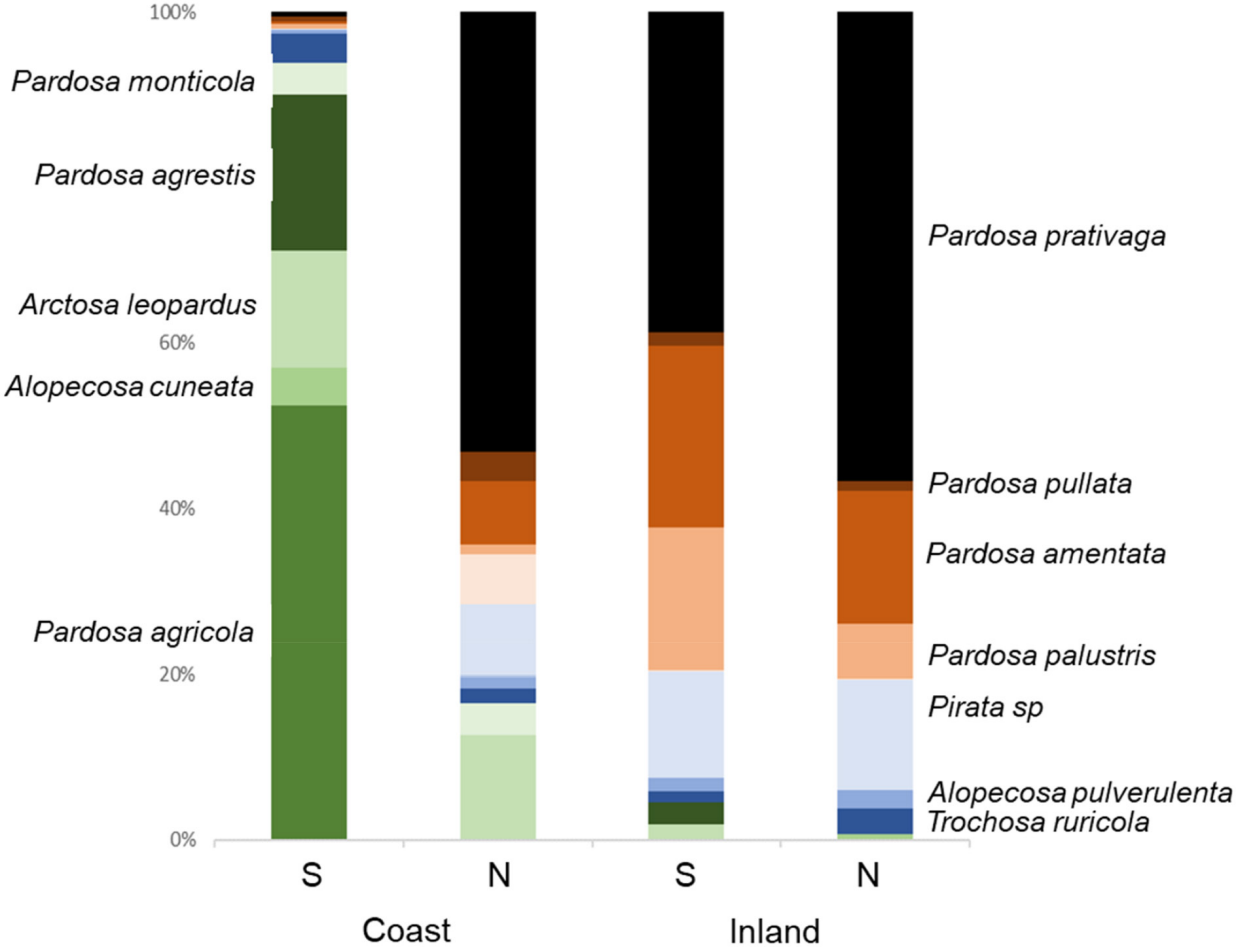
Relative abundance of wolf spider species in inland and coastal sites in Halland/Kalmar (S) and in Uppland (N).

The analysis of spider communities included 3931 spider individuals separated into 16 taxa (Figure 2). The variation in community composition was explained by a region‐by‐inland/coast interaction (Wald statistics = 8.3, *p* < .001) and not by the presence or absence of a thick wrack bed (Wald statistics = 4.7, *p* > .1). The region‐by‐inland/coast interaction arose because of a larger difference between southern and northern coastal sites compared with southern and northern inland sites (Figure 2). When comparing abundances at the species level (Table 1), four species (*Pardosa agrestis*, *P. agricola, Arctosa leopardus*, and *Alopecosa cuneata*) were found almost exclusively at southern coastal sites and three taxa (*Pardosa prativaga, P. amentata*, and *Pirata* spp. [mainly *P. piraticus*]) almost never occurred in these sites but were abundant elsewhere (Figure 2). In addition, one species (*P. monticola*) was mainly coastal whereas another species (*Pardosa palustris*) occurred mainly inland, irrespective of region.

**TABLE 1:** Marginal deviance and adjusted significances for the abundance of wolf spider species relative to coast/inland (CI), region (R, north [Uppland]/south [Kalmar/Halland]), and the CI‐by‐R interaction (ns = nonsignificant [*p* > .1])

Species	CI	R	CI*R
*Alopecosa cuneata*	1.4 ns	0.3 ns	10.4 (*p* < .02)
*A. pulverulenta*	0.1 ns	2.9 ns	0.8 ns
*Arctosa leopardus*	24.5 (*p* < .001)	2.9 ns	8.1 (*p* < .05)
*Pardosa agrestis*	11.8 (*p* < .02)	25.8 (*p* < .001)	0.2 ns
*P. agricola*	25.9 (*p* < .001)	16.1 (*p* < .005)	0.0 ns
*P. amentata*	1.5 ns	1.0 ns	10.6 (*p* < .05)
*P. fulvata*	6.8 ns	3.5 ns	5.0 ns
*P. monticola*	42.7 (*p* < .001)	0.7 ns	0.0 ns
*P. palustris*	13.5 (*p* < .005)	0.8 ns	5.6 ns
*P. prativaga*	0.4 ns	14.6 (*p* < .006)	36.6 (*p* < .001)
*P. pullata*	2.1 ns	0.7 ns	3.2 ns
*Pirata* spp.	1.1 ns	4.6 ns	13.1 (*p* < .007)
*Trochosa ruricola*	3.0 ns	1.7 ns	1.7 ns
*T. terricola*	2.1 ns	19.7 (*p* < .001)	0.0 ns

**FIGURE 3:**
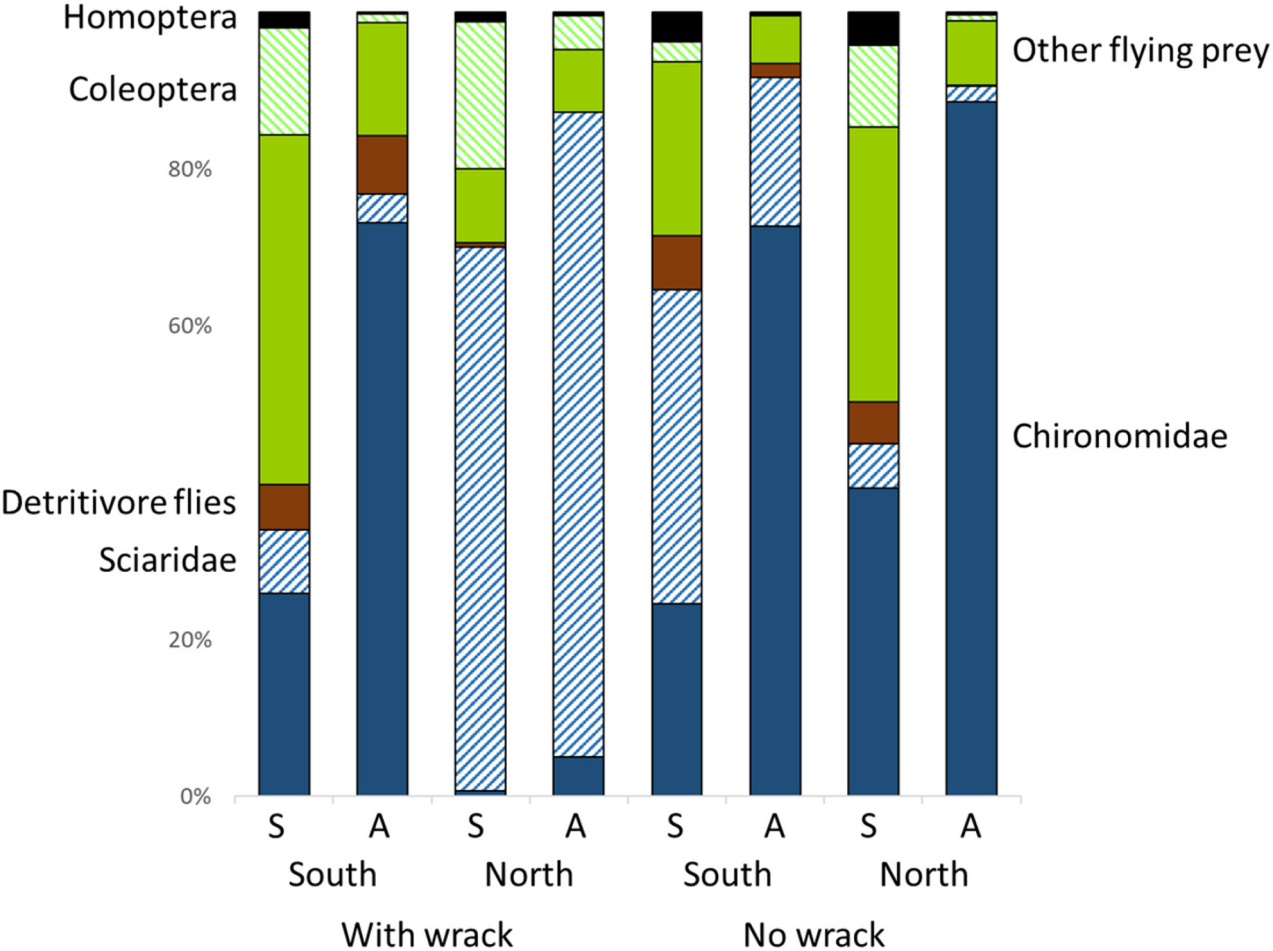
Relative abundances of prey catches in SLAM traps, separated by wrack occurrence, region (south = Kalmar, north = Uppland), and season (A = August, S = July). Detritivore flies include Sepsidae, Sphaeroceridae, and Coelopidae. Other flying prey include Hymenoptera and Lepidoptera but also a range of terrestrial Diptera.

The variation in the prey community was explained by a region‐by‐wrack interaction (Wald statistics = 11.4, *p* < .001) and by season (Wald statistics = 11.6, *p* < .002) (Figure 3). The region‐by‐wrack interaction occurred because Coleoptera (Deviance = 14.1, *p* < .02) and Sciaridae (Deviance = 20.3, *p* < .003) were positively affected by wrack availability only in northern sites, whereas Dolichopodidae (Deviance = 11.5, *p* < .05) was negatively affected by wrack availability only in southern sites (Figure 3). The seasonal effect occurred because Empididae (Deviance = 12.4, *p* < .04) and Homoptera (Deviance = 12.8, *p* < .03) were more abundant during the early season in June, whereas Chironomidae (Deviance = 14.7, *p* < .03) and Trichoptera (Deviance = 12.5, *p* < .04) were more abundant during August.

**FIGURE 4:**
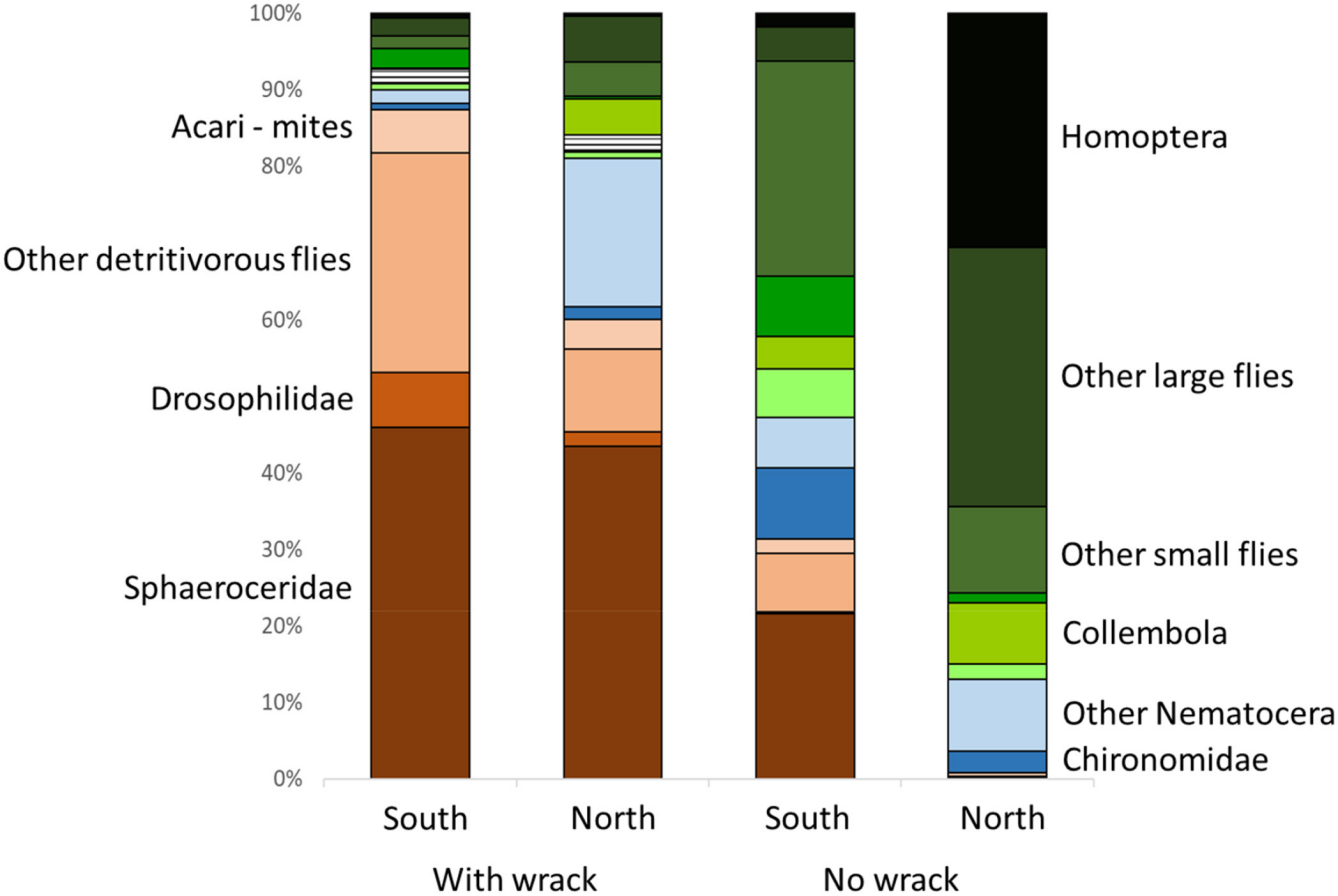
Relative contents of spider guts from sites with or without wrack and in the northern (Uppland) or southern (Kalmar) region. Other detritivore flies include those connected to wracksf, such as Anthomyiidae, Coelopidae, Ephydridae, and Sepsidae. Other large flies include Dolichopodidae, Dryomyzidae, Empididae, Fannidae, Heleomyzidae, Hybotidae, Muscidae, Rhinophoridae, Scatophagidae, Sciomyzidae, Syrphidae, Tabanidae, and Tachinidae. Other small flies include Acroceridae, Agromyzidae, Asteiidae, Canacidae, Carnidae, Chamaemyiidae, Chloropidae, Lonchopteridae, Opomyzidae, Phoridae, and Pipunculidae. Other Nematocera include Cecidomyiidae, Ceratopogonidae, Keroplatidae, Limoniidae, Mycetophilidae, Psychodidae, Scatopsidae, and Sciaridae.

The number of prey items encountered in the gut of spider individuals varied between one and 15, with an average of 3.9. The dominant order in the guts was Diptera, both Brachycera (60%) and Nematocera (18%), with minor amounts of other groups; Homoptera (10%, mainly Cicadellidae and Delphacidae), Collembola (4%), other flying prey (3%, Hymenoptera and Lepidoptera), Formicidae (2%), Acari (2%), and Heteroptera (1%) (Figure 4, Table A2). The diet contents varied considerably among sites and were mainly explained by wrack (Lawley‐Hotelling trace statistics = 6.1, *p* < .001) and season (Lawley‐Hotelling trace statistics = 2.6, *p* < .003), with an almost significant effect from a region‐by‐wrack interaction (Lawley‐Hotelling trace statistics = 1.7, *p* < .07) (Figure 4). However, there was no effect of spider species either when including this variable alone or in combination with other variables, or when run separately for the region. Because of the almost significant region‐by‐wrack interaction on gut contents, we repeated the analysis for sites with or without wrack separately. In this analysis, the region was significant for sites without wrack (*p* < .03) but not for sites with wrack (*p* > .2). The prey groups explaining the region difference for sites without wrack were Sphaeroceridae (*F*_1,6_ = 141, *p* < .003) and Enchytraeidae (*F*_1,6_ = 111, *p* < .05); that both had a higher frequency in spider guts from southern sites (Figure 4). Finally, the comparison between sites with or without wrack suggested that mainly Sphaeroceridae (*F*_1,11_ = 17.2, *p* < .06) and Heteroptera (*F*_1,11_ = 19.9, *p* < .05) were more abundant in spider guts from the site with wrack.

**FIGURE 5:**
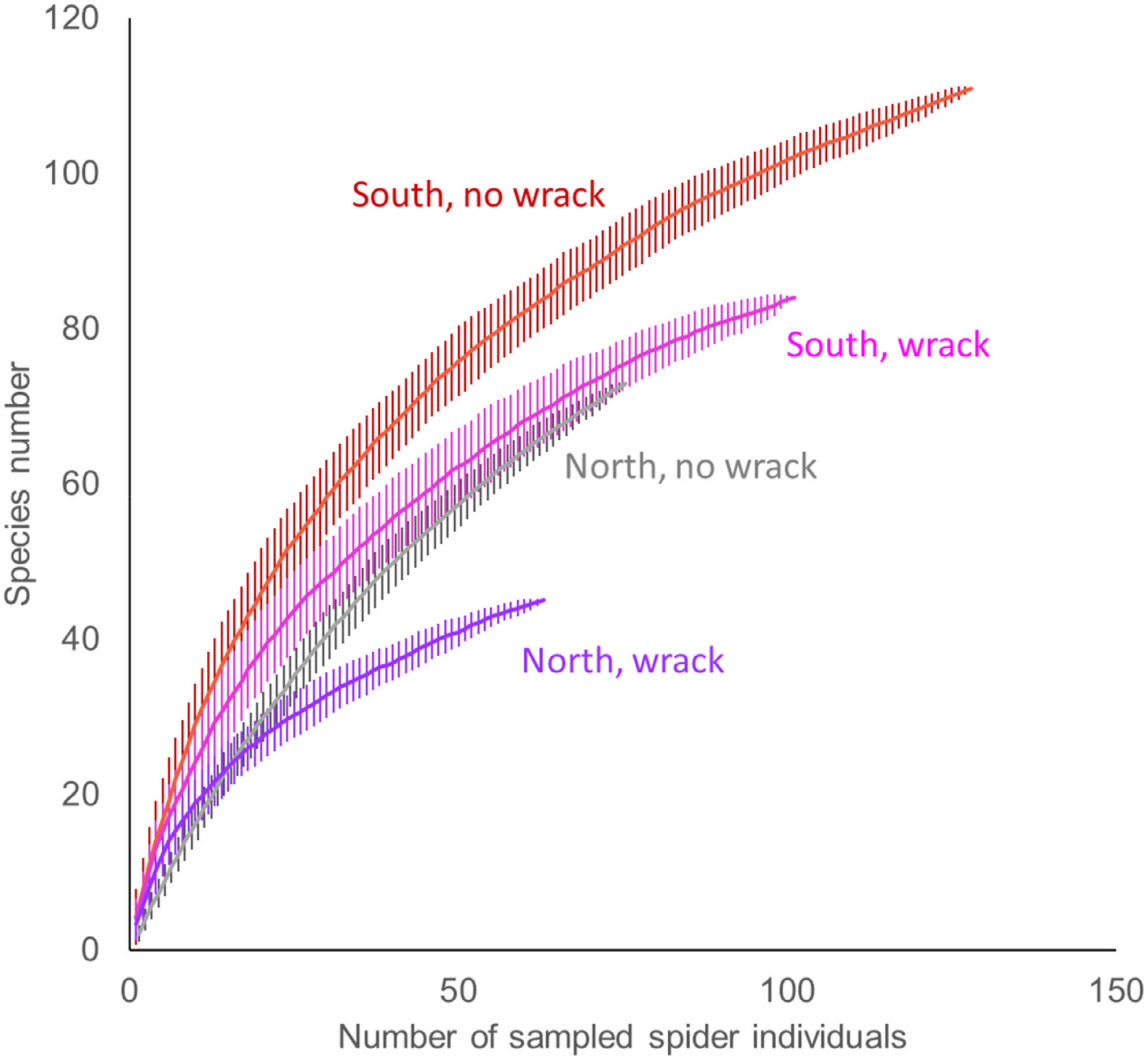
Species accumulation curves (±SD) relative to the number of sampled spiders for northern sites (Uppland) and southern sites (Kalmar), with or without wrack accumulation.

**FIGURE 6:**
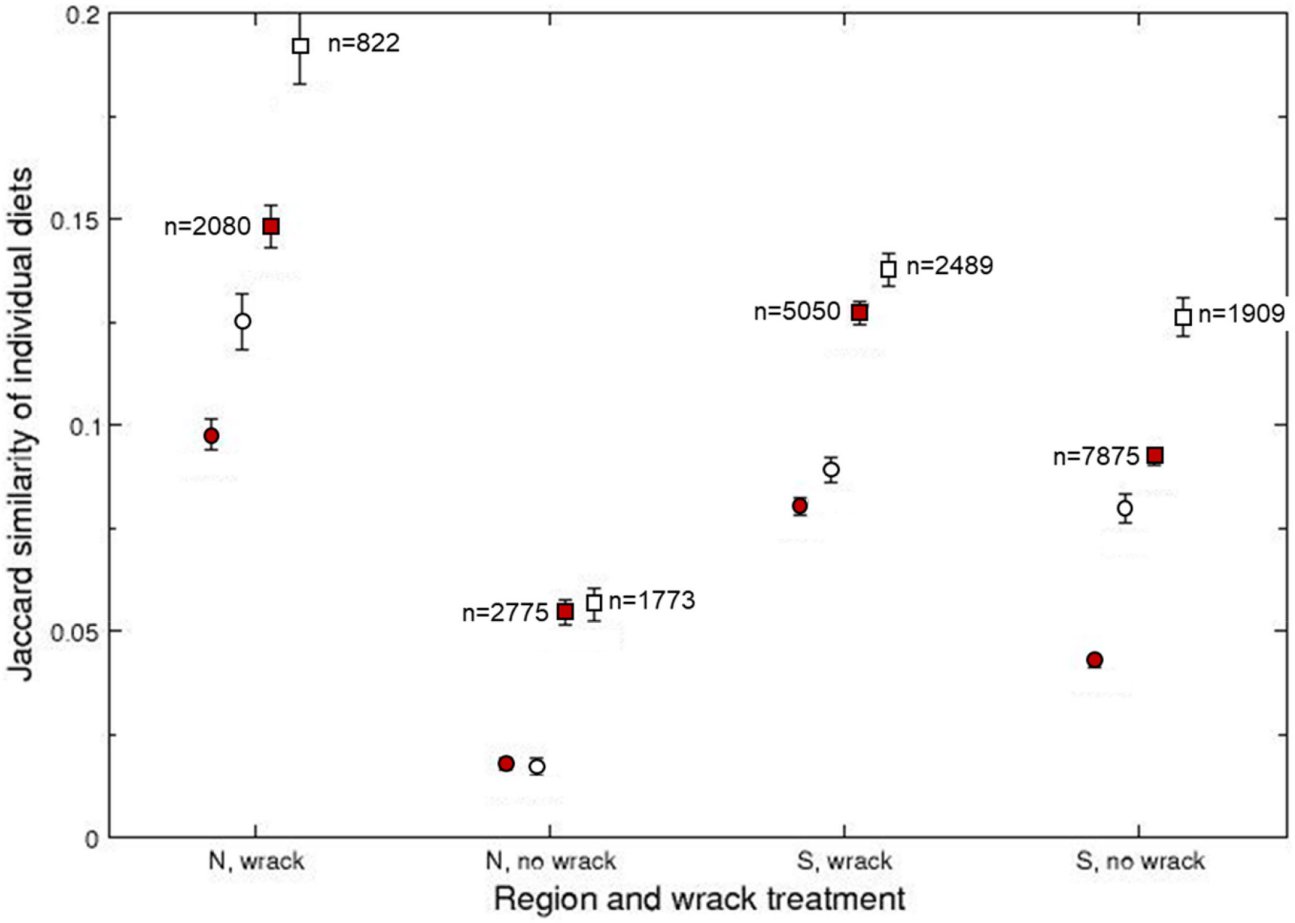
Individual diet similarity estimated as Jaccard similarity index (±SE) separated for the region (N=Uppland, S=Kalmar) and wrack presence. The diet similarity was estimated between all pairs of individuals (red) or between pairs of the same species (white) and when prey were included at the species (circles) or family (squares) level. N‐values refer to the number of pairs of individuals.

The species accumulation curves indicated that prey diversity was higher in southern sites and in sites with no wrack compared with northern sites and wrack sites (Figure 5). When comparing diet consistency, we found that individual spiders had, on average, a Jaccard similarity of diets = 0.056 (sharing approximately 5.6% of the prey species consumed by two individuals). Diet consistency between any pair of individual spiders varied with region (β =0.025, *p* < .001), wrack (β = 0.080, *p* < .001), and their interaction (β = −0.042, *p* < .001). The interaction arose because spider from northern wrack sites had higher diet consistency than spiders from southern wrack sites, whereas spiders from northern nonwrack sites had lower consistency than those from southern nonwrack sites (Figure 6). These diet similarities were larger when performed for pairs of the same spider species (Figure 6), but patterns were otherwise similar (region: β = 0.062, *p* < .001; wrack: β = 0.108, *p* < .001; interaction: β = −0.099, *p* < .001) and when diets were estimated at the prey family level both for all spider individuals (region: β = 0.038, *p* < .001; wrack: β = 0.094, *p* < .001; interaction: β = −0.059, *p* < .001) and for pairs of the same spider species (region: β = 0.070, *p* < .001; wrack: β = 0.136, *p* < .001; interaction: β = −0.124, *p* < .001).

## 4 DISCUSSION

The spider community showed large regional changes along the Baltic Sea seashore despite comparatively small salinity differences (5 vs. 7‰). Several spider species (*Pardosa agrestis*, *P. agricola, Arctosa leopardus*, and *Alopecosa cuneata*) were almost exclusively located on the higher salinity sites compared with lower salinity sites by the Baltic Sea shore and by inland lakeshores. At the same time, other taxa (*Pardosa prativaga, P. amentata*, and *Pirata* spp.) had the opposite distribution pattern, and this pattern was seemingly not explained by either prey availability or actual spider diets. In fact, there were no detectable diet differences between spider species or between spiders captured on shores with different salinity levels. Instead, spider diets varied between shores with or without thick beds of stranded wrack, a gradient that did not affect spider community structure. Consequently, and because the species shift only occurred on coastal sites and not on corresponding inland sites, it seems that coastal spider communities are directly affected by the saline conditions.

High salinity has several negative impacts on spiders and other arthropods, by reducing both survival and reproduction (Foucreau et al., 2012; Pétillon et al., 2011; Puzin et al., 2011). Even though none of the species found on the Baltic shorelines can be considered true halophilic and are usually not found on more marine seashores (Pétillon et al., 2008), it seems reasonable to assume that species vary in their sensitivity to saline conditions. However, please note that previous studies on wolf spiders tested the responses of individuals at much higher salinity (>30‰) than in our sites, and it is unclear to what extent that their conclusions could be extrapolated to our study. Irrespective of the mechanisms, our data in combination with previous studies suggest a gradient in salinity thresholds of the dominant wolf spider species on marine shorelines in northwestern Europe where *P. prativaga* typically dominates low salinity sites, *P. agricola* dominates intermediate salinity sites, and *P. purbeckensis* dominates high salinity sites. The species abundance distributions of wolf spider communities are often highly skewed with one dominant species having more than 60% of all individuals and a tail of rare species. Even though low salinity sites are not always dominated by *P. prativaga*, two‐thirds are dominated by this species and then more rarely by *P. amentata*, *P. palustris*, and some other species (see also Meriste et al., 2016).

Whereas the restriction to low salinity sites can likely be explained by salt sensitivity, the corresponding absence of other species at the same low salinity sites seems more puzzling. First, it is evident that the absence from low salinity sites is not absolute as both *Pardosa agrestis* and *P. agricola* are frequently reported also from inland habitats in central Europe and more rarely from inland sites in northern Europe (GBIF.org). Moreover, studies on *P. purbeckensis*, perhaps the most halophilic species, suggest that fitness is not reduced on low salinity sites (Pétillon et al., 2011). It is possible that some other habitat characteristics restrict their occurrence at low salinity sites or that distributions are restricted by species interactions. Several wolf spider species are known for intraguild predation of other wolf spider species, at least in the laboratory, and dominance is mainly governed by size differences (Buddle et al., 2003; Rickers et al., 2006; Rypstra et al., 2007; Rypstra & Samu, 2005; Turney & Buddle, 2019), but no study this far has evaluated the role of intraguild predation on the spatial distribution of wolf spiders.

Whatever the reason is for the difference in wolf spider community composition, the patterns are not likely explained by different dietary niches among spider species or by differences in prey availability. Both this and previous studies using either molecular gut content analysis or other methods indicate large overlaps in the diet of wolf spider species (Mellbrand & Hambäck, 2010; Verschut et al., 2019). Diet differences observed in this study instead seem to depend on whether spiders were collected on sites with or without accumulated wrack, but these diet shifts did not coincide with shifts in the wolf spider community. By far the most abundant prey group in the wolf spider guts on sites with either wrack or no wrack were dipterans (typically taxa with smaller individuals) and to some extent homopterans. This general prey composition of wolf spiders is of course well‐known from nonmolecular studies (e.g., Nyffeler, 1999), but the relative importance of small dipterans is perhaps larger in our study habitats. Some differences between molecular and nonmolecular studies may occur because the former provides an improved representation of small prey items, which are easily overlooked in nonmolecular studies due to more rapid consumption. In either case, wolf spiders are likely quite opportunistic predators where prey choice perhaps depends more on encounter probabilities and catchability of prey in their selected habitat than on prey qualities. This opportunistic behavior is perhaps also reflected in the different number of prey species, where the number is higher in southern sites, as expected, and in sites with no wrack. Similarly, diet consistency was also higher on wrack sites, and both patterns observed for wrack sites may reflect that wrack beds are dominated by a small set of detritivorous species. More surprising was the higher diet consistency of spiders on southern nonwrack sites compared with northern nonwrack sites, despite the lower total prey diversity observed for the spiders in the southern region.

Even though opportunism seems to be a dominant pattern, particularly dark‐winged fungus gnats (Sciaridae) are underrepresented in wolf spider guts despite their comparatively high occurrence at these sites, similar to what was found previously (Verschut et al., 2019). The reason for spiders to avoid fungus gnats may be that they represent low‐quality food (as suggested by Toft & Wise, 1999a, 1999b). Diet differences between sites with or without accumulated wrack otherwise reflect availability, even though we refrained from testing the availability‐use relationship due to the bias in SLAM trap catches. Many small flies often occurring on wrack beds, such as Drosophilidae, Ephydridae, Sepsidae, and Sphaeroceridae are underrepresented in Malaise‐type traps on shorelines because these flies tend to stick to the ground. In either case, these small detritivorous flies that likely developed in or close to the decomposing wrack made up more than 75% of all prey in spider guts when collected from sites with heavy wrack beds, and the diet composition was surprisingly similar for spiders collected on northern and southern wrack beds. More unexpected was perhaps the low frequency of chironomids in the spider gut contents, particularly in the nonwrack sites. In a previous study (Verschut et al., 2019), not far from the sites included in this paper, chironomids dominated the spider gut contents and particularly late in the season. In this study, there were no seasonal differences, and spiders on nonwrack sites instead consumed a range of terrestrial prey groups, such as Homoptera and various terrestrial Diptera (Chloropidae, Empididae, Dolichopodidae etc.), and it seems that spiders were less strongly connected to the nearby marine environment than previously assumed. In either case, this variability among studies indicates how dynamic the food choice of spiders may be.

To summarize, our study indicates that quite a small difference in salinity caused the species composition of wolf spider communities to change almost completely. The mechanism underlying this community shift is less obvious, why species disappear either in the high salinity or in the low salinity ends, but we can conclude that prey availability or differences in the trophic niche between species is likely not involved.

## AUTHOR CONTRIBUTIONS

**Peter A Hambäck:** Conceptualization (lead); data curation (lead); formal analysis (lead); funding acquisition (lead); investigation (supporting); methodology (supporting); project administration (lead); resources (lead); software (lead); supervision (lead); visualization (lead); writing – original draft (lead). **Alyssa R. Cirtwill:** Conceptualization (supporting); investigation (supporting); visualization (supporting); writing – review and editing (supporting). **Magdalena Grudzinska‐Sterno:** Investigation (equal); writing – review and editing (supporting). **Alexander Hoffmann:** Investigation (equal). **Marie Langbak:** Investigation (equal). **David Åhlén:** Investigation (equal); writing – review and editing (supporting).

## CONFLICT OF INTEREST

The authors have no competing financial or personal interests that would conflict with the content of this paper.

## Data Availability

The data that support the findings of this study are openly available in Dryad at http://doi.org/10.5061/dryad.gxd2547qk (Hambäck et al., 2023).

## Appendix

**TABLE A1:** Site information, where spider communities were quantified in all sites and where diet data and prey availabilities were collected from a subset of sites

Site	Coast/inland	Region	Wrack	Diet data	Latitude	Longitude
Barnens Ö	Coast	Uppland	Y	Y	59° 55′ 38″ N	18° 56′ 29″ E
Forsmark	Coast	Uppland	N	Y	60° 23′ 41″ N	18° 13′ 12″ E
Gudinge	Coast	Uppland	N		60° 30′ 31″ N	17° 59′ 46″ E
Klungsten	Coast	Uppland	N	Y	60° 32′ 44″ N	18° 1′ 10″ E
Raggarön	Coast	Uppland	N		60° 12′ 18″ N	18° 33′ 55″ E
Rådmansholmen	Coast	Uppland	N		59° 37′ 18″ N	18° 56′ 52″ E
Senneby Hake	Coast	Uppland	Y	Y	59° 58′ 23″ N	18° 54′ 21″ E
Skedvik	Coast	Uppland	N		58° 58′ 15″ N	17° 45′ 1″ E
Storsten	Coast	Uppland	N	Y	60° 31′ 16″ N	18° 0′ 23″ E
Sveden	Coast	Uppland	N		59° 35′ 12″ N	18° 39′ 58″ E
Tranviken	Coast	Uppland	N		60° 9′ 52″ N	18° 46′ 43″ E
Tullgarn	Coast	Uppland	N		58° 57′ 22″ N	17° 35′ 10″ E
Tvärnö	Coast	Uppland	N	Y	60° 12′ 9″ N	18° 32′ 42″ E
Bergianska	Inland	Uppland	N		59° 22′ 3″ N	18° 2′ 46″ E
Frötuna	Inland	Uppland	N		59° 54′ 45″ N	17° 51′ 52″ E
Jönsbolsjön	Inland	Uppland	N		59° 51′ 21″ N	18° 1′ 52″ E
Kromsta	Inland	Uppland	N		59° 40′ 38″ N	17° 15′ 13″ E
Kärven	Inland	Uppland	N		59° 54′ 54″ N	18° 9′ 10″ E
Ludden	Inland	Uppland	N		59° 46′ 18″ N	18° 40′ 23″ E
Ribbingebäck	Inland	Uppland	N		59° 50′ 17″ N	17° 10′ 54″ E
Segersta viltvatten	Inland	Uppland	N		59° 38′ 12″ N	17° 24′ 27″ E
Senneby	Inland	Uppland	N		59° 57′ 47″ N	18° 51′ 13″ E
Örsundsbro	Inland	Uppland	N		59° 44′ 47″ N	17° 20′ 47″ E
U27	Inland	Uppland	N		60° 12′ 59″ N	18° 12′ 38″ E
U28	Inland	Uppland	N		60° 12′ 39″ N	18° 15′ 41″ E
U46	Inland	Uppland	N		59° 54′ 46″ N	17° 23′ 45″ E
U113	Inland	Uppland	N		59° 41′ 11″ N	17° 54′ 19″ E
U132	Inland	Uppland	N		59° 27′ 41″ N	17° 41′ 56″ E
Dösjön	Coast	Kalmar	N	Y	56° 28′ 35″ N	16° 8′ 39″ E
Enetri	Coast	Kalmar	N	Y	56° 15′ 7″ N	16° 29′ 8″ E
Enudden	Coast	Kalmar	N	Y	56° 30′ 59″ N	16° 11′ 13″ E
Fågelmara	Coast	Kalmar	N	Y	56° 13′ 28″ N	16° 0′ 53″ E
Grisbäck	Coast	Kalmar	N	Y	56° 19′ 59″ N	16° 4′ 10″ E
Sandvik	Coast	Kalmar	Y	Y	56° 22′ 22″ N	16° 24′ 14″ E
Ventlinge	Coast	Kalmar	Y	Y	56° 17′ 2″ N	16° 23′ 49″ E
BA6	Inland	Halland	N		56° 34′ 60″ N	13° 4′ 5″ E
BA700	Inland	Halland	N		56° 34′ 52″ N	13° 5′ 27″ E
D2	Inland	Halland	N		56° 38′ 25″ N	12° 59′ 49″ E
D3	Inland	Halland	N		56° 37′ 58″ N	13° 0′ 15″ E
D7	Inland	Halland	N		56° 36′ 34″ N	12° 55′ 28″ E
D12	Inland	Halland	N		56° 26′ 7″ N	12° 56′ 30″ E
D15	Inland	Halland	N		56° 26′ 52″ N	13° 0′ 37″ E
D16	Inland	Halland	N		56° 27′ 0″ N	13° 3′ 41″ E
D17	Inland	Halland	N		56° 40′ 5″ N	12° 46′ 46″ E
D18	Inland	Halland	N		56° 38′ 40″ N	12° 47′ 19″ E
D19	Inland	Halland	N		56° 45′ 28″ N	12° 39′ 43″ E
D20	Inland	Halland	N		56° 44′ 15″ N	12° 46′ 4″ E
D22	Inland	Halland	N		56° 45′ 3″ N	12° 52′ 52″ E
D23	Inland	Halland	N		56° 49′ 47″ N	12° 53′ 28″ E
D25	Inland	Halland	N		56° 52′ 13″ N	12° 45′ 31″ E
D28	Inland	Halland	N		56° 52′ 44″ N	12° 44′ 57″ E
D29	Inland	Halland	N		56° 57′ 55″ N	12° 24′ 15″ E
EA16	Inland	Halland	N		56° 27′ 44″ N	13° 4′ 27″ E
EA18	Inland	Halland	N		56° 27′ 40″ N	13° 5′ 13″ E
EA60	Inland	Halland	N		56° 27′ 44″ N	13° 8′ 43″ E
KA7	Inland	Halland	N		56° 49′ 23″ N	12° 39′ 51″ E
MA18	Inland	Halland	N		56° 25′ 38″ N	13° 5′ 24″ E
VA4	Inland	Halland	N		56° 33′ 45″ N	13° 6′ 26″ E

**TABLE A2:** Total list of prey species with DNA in spider guts, and the number of spiders where the prey DNA was detected

Prey order	Family	Species	No of spiders
Annelidae	Enchytraeidae	*Enchytraeus albidus*	6
Annelidae	Enchytraeidae	*Enchytraeus buchholzi*	1
Annelidae	Enchytraeidae	*Enchytraeus moebii/albidus*	4
Annelidae	Enchytraeidae	*Lumbricillus pagenstecheri*	3
Aranae	Clubionidae	*Clubiona phragmitis*	3
Aranae	Clubionidae	*Clubiona reclusa/norwegica*	2
Aranae	Linyphiidae	*Erigone arctica*	17
Aranae	Linyphiidae	*Erigone longipalpis*	7
Aranae	Theridiidae	*Anelosimus vittatus*	1
Aranae	Theridiidae	*Theridion varians*	1
Coleoptera	Anthicidae	*Anthicus flavipes*	2
Coleoptera	Carabidae	*Bembidion pallidipenne*	2
Coleoptera	Carabidae	*Elaphrus uliginosus*	1
Coleoptera	Carabidae	*Pterostichus rhaeticus/nigrita*	1
Coleoptera	Carabidae	*Trechus secalis*	2
Coleoptera	Chrysomelidae	*Chrysolina staphylaea*	2
Coleoptera	Curculionidae	*Pelenomus quadrituberculatus*	6
Coleoptera	Dermestidae	*Dermestes szekessyi*	5
Coleoptera	Hydraenidae	*Ochthebius marinus/minimus*	1
Coleoptera	Hydrophilidae	*Cercyon depressus*	3
Coleoptera	Scarabaeidae	*Melolontha melolontha*	1
Coleoptera	Scarabaeidae	*Serica brunnea*	1
Coleoptera	Silphidae	*Oiceoptoma thoracicum*	2
Coleoptera	Staphylinidae	*Acrolocha sulcula*	1
Coleoptera	Staphylinidae	*Aleochara bipustulata/verna*	2
Coleoptera	Staphylinidae	*Atheta fungi*	1
Coleoptera	Staphylinidae	*Atheta vestita*	3
Coleoptera	Staphylinidae	*Cafius xantholoma*	1
Coleoptera	Staphylinidae	*Carpelimus rivularis*	18
Coleoptera	Staphylinidae	*Cordalia obscura*	1
Coleoptera	Staphylinidae	*Gnypeta carbonaria*	4
Coleoptera	Staphylinidae	*Omalium riparium*	9
Coleoptera	Staphylinidae	*Tachyporus nitidulus*	1
Collembola	Entomobryidae	*Desoria grisea*	8
Collembola	Entomobryidae	*Entomobrya lanuginosa*	2
Collembola	Entomobryidae	*Entomobrya multifasciata*	3
Collembola	Entomobryidae	*Lepidocyrtus lignorum*	1
Collembola	Entomobryidae	*Orchesella cincta/villosa*	5
Collembola	Entomobryidae	*Orchesella flavescens*	2
Collembola	Hypogastruridae	*Hypogastrura viatica*	2
Collembola	Isotomidae	*Halisotoma maritima*	9
Collembola	Isotomidae	*Isotoma anglicana*	3
Collembola	Isotomidae	*Isotoma riparia*	31
Collembola	Isotomidae	*Isotoma viridis/coerulea*	8
Collembola	Isotomidae	*Isotomurus fucicolus*	3
Collembola	Katiannidae	*Sminthurinus signatus*	1
Collembola	Sminthuridae	*Sminthurinus aureus*	2
Collembola	Sminthuridae	unid	2
Collembola	Tomoceridae	*Pogonognathellus* spp	2
Diptera	Acroceridae	*Acrocera orbicula*	16
Diptera	Acroceridae	*Ogcodes pallipes*	3
Diptera	Agromyzidae	*Agromyza albipennis*	1
Diptera	Agromyzidae	*Agromyza filipendulae*	1
Diptera	Agromyzidae	*Phytomyza horticola*	6
Diptera	Anthomyiidae	*Delia florilega*	1
Diptera	Anthomyiidae	*Delia platura*	10
Diptera	Anthomyiidae	*Fucellia fucorum*	31
Diptera	Anthomyiidae	*Fucellia tergina/maritima*	36
Diptera	Asteiidae	*Asteia amoena*	1
Diptera	Carnidae	*Meoneura* sp.	1
Diptera	Cecidomyiidae	*Rhopalomyia* sp.	4
Diptera	Cecidomyiidae	unid	26
Diptera	Ceratopogonidae	*Atrichopogon fusculus*	4
Diptera	Ceratopogonidae	*Bezzia annulipes*	4
Diptera	Ceratopogonidae	*Culicoides newsteadi*	1
Diptera	Ceratopogonidae	*Dasyhelea turficola*	12
Diptera	Ceratopogonidae	*Forcipomyia hygrophila*	1
Diptera	Ceratopogonidae	*Palpomyia lineata*	2
Diptera	Ceratopogonidae	unid	2
Diptera	Chamaemyiidae	*Chamaemyia geniculata*	2
Diptera	Chironomidae	*Arctopelopia griseipennis*	1
Diptera	Chironomidae	*Chironomus aprilinus/pseudothummi*	6
Diptera	Chironomidae	*Cladopelma virescens*	1
Diptera	Chironomidae	*Cladotanytarsus difficilis*	1
Diptera	Chironomidae	*Cladotanytarsus gedanicus*	4
Diptera	Chironomidae	*Cladotanytarsus mancus*	20
Diptera	Chironomidae	*Cladotanytarsus nigrovittatus*	2
Diptera	Chironomidae	*Cladotanytarsus wexionensis/bicornutus*	1
Diptera	Chironomidae	*Cricotopus caduceus/patens/flavocinctus*	11
Diptera	Chironomidae	*Cricotopus ornatus*	1
Diptera	Chironomidae	*Halocladius variabilis*	2
Diptera	Chironomidae	*Limnophyes* sp.	1
Diptera	Chironomidae	*Metriocnemus atriclava*	1
Diptera	Chironomidae	*Paratanytarsus inopertus/Chironomus plumosus*	16
Diptera	Chironomidae	*Paratanytarsus natvigi*	8
Diptera	Chironomidae	*Psectrocladius limbatellus*	4
Diptera	Chironomidae	*Psectrocladius oxyura*	10
Diptera	Chironomidae	*Pseudosmittia trilobata*	3
Diptera	Chironomidae	*Smittia leucopogon*	1
Diptera	Chironomidae	*Smittia* sp.	1
Diptera	Chironomidae	*Tanytarsus gracilentus*	1
Diptera	Chironomidae	*Tanytarsus usmaensis*	5
Diptera	Chironomidae	unid	1
Diptera	Chloropidae	*Chlorops pumilionis*	1
Diptera	Chloropidae	*Eutropha fulvifrons*	4
Diptera	Chloropidae	*Incertella* sp.*/Rhopalopterum* sp.	3
Diptera	Chloropidae	*Meromyza nigriventrix/saltatrix*	1
Diptera	Chloropidae	*Oscinella* sp.	56
Diptera	Chloropidae	*Thaumatomyia notata*	2
Diptera	Coelopidae	*Coelopa frigida*	17
Diptera	Dolichopodidae	*Dolichopus nitidus*	1
Diptera	Dolichopodidae	*Dolichopus nubilus*	20
Diptera	Dolichopodidae	*Dolichopus pumilus and related species*	4
Diptera	Dolichopodidae	*Gymnopternus aerosus*	3
Diptera	Dolichopodidae	*Medetera truncorum/petrophiloides*	1
Diptera	Dolichopodidae	*Syntormon pallipes*	1
Diptera	Dolichopodidae	*Xanthochlorus ornatus*	1
Diptera	Drosophilidae	*Cacoxenus argyreator*	4
Diptera	Drosophilidae	*Scaptomyza flava/pallida*	36
Diptera	Dryomyzidae	*Heterocheila buccata*	8
Diptera	Empididae	*Rhamphomyia geniculata*	3
Diptera	Empididae	unid	2
Diptera	Ephydridae	*Discocerina obscurella*	5
Diptera	Ephydridae	*Hydrellia griseola*	1
Diptera	Ephydridae	*Lamproscatella sibilans*	3
Diptera	Ephydridae	*Limnellia quadrata*	1
Diptera	Ephydridae	*Paracoenia fumosa*	6
Diptera	Ephydridae	*Philotelma alaskense/defectum*	2
Diptera	Ephydridae	*Psilopa nigritella*	1
Diptera	Ephydridae	*Scatella paludum*	7
Diptera	Ephydridae	*Scatella stagnalis and related species*	23
Diptera	Ephydridae	*Scatella subguttata*	3
Diptera	Ephydridae	*Scatophila despecta*	2
Diptera	Heleomyzidae	*Trixoscelis obscurella*	3
Diptera	Keroplatidae	*Pyratula zonata*	1
Diptera	Limoniidae	*Symplecta stictica*	5
Diptera	Limoniidae	unid	1
Diptera	Lonchopteridae	*Lonchoptera bifurcata*	3
Diptera	Muscidae	*Coenosia lacteipennis*	1
Diptera	Muscidae	*Coenosia pedella/testacea*	6
Diptera	Muscidae	*Coenosia pumila*	1
Diptera	Muscidae	*Coenosia testacea*	1
Diptera	Muscidae	*Lispocephala erythrocera*	1
Diptera	Muscidae	*Morellia sinensis/tempestiva*	1
Diptera	Muscidae	*Schoenomyza litorella*	1
Diptera	Muscidae	*Spilogona aerea*	1
Diptera	Opomyzidae	*Geomyza* sp.	1
Diptera	Opomyzidae	*Opomyza germinationis/florum*	2
Diptera	Phoridae	*Megaselia albicans*	2
Diptera	Phoridae	*Megaselia brevicostalis*	14
Diptera	Phoridae	*Megaselia manicata*	4
Diptera	Phoridae	*Megaselia pleuralis*	5
Diptera	Phoridae	*Megaselia pusilla/ignobilis*	1
Diptera	Phoridae	*Megaselia* sp.	1
Diptera	Phoridae	*Metopina* sp.	3
Diptera	Phoridae	unid	5
Diptera	Pipunculidae	*Eudorylas fuscipes*	2
Diptera	Psychodidae	*Psychoda lativentris*	2
Diptera	Rhinophoridae	*Tricogena rubricosa*	2
Diptera	Scathophagidae	*Spaziphora hydromyzina*	1
Diptera	Scathophagidae	*Trichopalpus fraternus*	2
Diptera	Scatopsidae	*Coboldia fuscipes*	21
Diptera	Scatopsidae	*Scatopse notata*	10
Diptera	Sciaridae	*Corynoptera inundata*	1
Diptera	Sciaridae	*Lycoriella sativae*	13
Diptera	Sciomyzidae	*Ditaeniella grisescens*	1
Diptera	Sepsidae	*Themira putris*	43
Diptera	Sphaeroceridae	*Coproica hirtula*	2
Diptera	Sphaeroceridae	*Coproica lugubris*	9
Diptera	Sphaeroceridae	*Leptocera curvinervis*	73
Diptera	Sphaeroceridae	*Opacifrons coxata*	5
Diptera	Sphaeroceridae	*Opalimosina mirabilis*	4
Diptera	Sphaeroceridae	*Pullimosina heteroneura*	6
Diptera	Sphaeroceridae	*Pullimosina pullula*	2
Diptera	Sphaeroceridae	*Rachispoda intermedia/fuscipennis*	34
Diptera	Sphaeroceridae	*Rachispoda limosa*	10
Diptera	Sphaeroceridae	*Rachispoda lutosa/breviceps*	10
Diptera	Sphaeroceridae	*Thoracochaeta seticosta*	58
Diptera	Sphaeroceridae	*Thoracochaeta zosterae*	51
Diptera	Syrphidae	*Eristalis* sp.	1
Diptera	Syrphidae	*Eupeodes corollae*	1
Diptera	Syrphidae	*Platycheirus* sp.	1
Diptera	Tabanidae	*Chrysops relictus/viduatus/rufipes*	2
Diptera	Tabanidae	*Tabanus cordiger/unifasciatus*	1
Diptera	Tachinidae	*Siphona* sp.	3
Hemiptera	Anthocoridae	*Orius majusculus/minutus*	2
Hemiptera	Anthocoridae	*Orius niger/horvathi*	3
Hemiptera	Aphididae	*Euceraphis betulae*	1
Hemiptera	Cicadellidae	*Arthaldeus striifrons*	3
Hemiptera	Cicadellidae	*Euscelis sordida*	3
Hemiptera	Cicadellidae	*Limotettix striola*	3
Hemiptera	Cicadellidae	*Paralimnus phragmitis*	2
Hemiptera	Cicadellidae	*Planaphrodes bifasciatus*	1
Hemiptera	Cicadellidae	*Psammotettix confinis/alienus*	14
Hemiptera	Cicadellidae	*Psammotettix nodosus/dubius*	1
Hemiptera	Cicadellidae	*Sarhoanus* sp.	2
Hemiptera	Delphacidae	*Delphax crassicornis*	2
Hemiptera	Delphacidae	*Euconomelus lepidus*	3
Hemiptera	Delphacidae	*Evacanthus interruptus*	1
Hemiptera	Delphacidae	*Javesella dubia/pellucida/forcipata*	12
Hemiptera	Delphacidae	*Unkanodes* sp.	13
Hemiptera	Gerridae	*Gerris thoracicus*	2
Hemiptera	Miridae	*Atractotomus mali/Phytocoris pini*	1
Hemiptera	Miridae	*Closterotomus norwegicus*	2
Hemiptera	Miridae	*Orthotylus* sp.	19
Hemiptera	Nepidae	*Nepa cinerea/rubra*	1
Hemiptera	Piesmatidae	*Parapiesma quadratum*	3
Hemiptera	Saldidae	*Saldula* sp.	5
Hymenoptera	Aphelinidae	*Aphelinus asychis*	2
Hymenoptera	Aphelinidae	*Aphelinus* sp.	1
Hymenoptera	Braconidae	*Aphaereta minuta*	1
Hymenoptera	Braconidae	*Dolichogenida* sp.	2
Hymenoptera	Braconidae	*Praon flavinode*	1
Hymenoptera	Braconidae	unid	1
Hymenoptera	Encyrtidae	*Copidosoma floridanum*	1
Hymenoptera	Eulophidae	*Diglyphus isaea*	1
Hymenoptera	Eulophidae	*Pediobius* sp.	2
Hymenoptera	Eulophidae	*Tamarixia pronomus*	1
Hymenoptera	Eulophidae	unid	1
Hymenoptera	Formicidae	*Lasius niger*	14
Hymenoptera	Formicidae	*Lasius* sp.	3
Hymenoptera	Formicidae	*Myrmica rubra*	9
Hymenoptera	Formicidae	*Myrmica ruginodis*	12
Hymenoptera	Ichneumonidae	*Cotesia vestalis*	1
Hymenoptera	Ichneumonidae	*Diadegma armillatum*	1
Hymenoptera	Ichneumonidae	*Diadegma fenestrale/nanus*	2
Hymenoptera	Ichneumonidae	*Homotropus signatus*	1
Hymenoptera	Mymaridae	*Anaphes* sp.	2
Hymenoptera	Pteromalidae	*Halticoptera aenea*	1
Hymenoptera	Pteromalidae	*Psilonotus adamas*	2
Hymenoptera	Pteromalidae	*Pteromalus semotus*	1
Hymenoptera	Pteromalidae	*Trichomalopsis* sp.	3
Hymenoptera	Pteromalidae	*Trichomalus* sp.	6
Hymenoptera	Pteromalidae	unid	5
Lepidoptera	Gelechiidae	*Monochroa tetragonella*	1
Lepidoptera	Gelechiidae	*Scrobipalpa obsoletella*	6
Lepidoptera	Glyphypterigidae	*Glyphipterix thrasonella*	1
Lepidoptera	Noctuidae	*Autographa gamma*	1
Lepidoptera	Plutellidae	*Plutella xylostella*	14
Lepidoptera	Psychidae	*Narycia duplicella*	1
Lepidoptera	Psychidae	*Psyche casta*	1
Mesostigmata	Ascidae	unid	1
Mesostigmata	Blattiscociidae	*Cheiroseius* sp.	9
Mesostigmata	Eviphipidae	unid	14
Mesostigmata	Macrocheliidae	unid	11
Mesostigmata	Parasitidae	*Pergamasus crassipes*	2
Mesostigmata	Parasitidae	unid	39
Opiliones	Phalangiidae	*Mitopus morio*	1
Orthoptera	Acrididae	*Chorthippus* spp.	5
Orthoptera	Acrididae	*Stethophyma grossum*	2
Orthoptera	Tetrigidae	*Tetrix subulata*	1
Sarcoptiformes	Ameronothridae	*Ameronothrus*	1
Sarcoptiformes	Ceratozetidae	*Trichoribates novus*	1
Sarcoptiformes	Crotoniidae	*Platynothrus thori*	1
Thysanoptera	Thripidae	unid	6
Trichoptera	Leptoceridae	*Oecetis ochracea*	1
Trombidiformes	Erythraeidae	*Balaustium*	6
Trombidiformes	Eupodidae	unid	3
Trombidiformes	Hydryphantidae	*Hydryphantes crassipalpis*	1

*Note*: Species id is provided as multiple species or genus level when the identity cannot be resolved (i.e., multiple species with sequence similarity >97%).
